# Supplementary material for: Genetic structure and historical and contemporary gene flow of Astyanaxmexicanus in the Gulf of Mexico slope: a microsatellite-based analysis
Source: PeerJ. 2021 Feb 25;9:e10784. doi: 10.7717/peerj.10784 (PMC7916531; doi:10.7717/peerj.10784)
Supplement: Supplemental Information 1 [file peerj-09-10784-s001.docx]

Table S1. Specimen tissue vouchers of every locations.

| **Voucher tissue** | **Species** | **Mitochondrial assignment** | **Location** | **Basin** |
| --- | --- | --- | --- | --- |
| C61-1 | Astyanax mexicanus | Astyanax mexicanus | Cuatro Ciénegas | Bravo River |
| C61-2 | Astyanax mexicanus | Astyanax mexicanus | Cuatro Ciénegas | Bravo River |
| C61-3 | Astyanax mexicanus | Astyanax mexicanus | Cuatro Ciénegas | Bravo River |
| C61-4 | Astyanax mexicanus | Astyanax mexicanus | Cuatro Ciénegas | Bravo River |
| C61-5 | Astyanax mexicanus | Astyanax mexicanus | Cuatro Ciénegas | Bravo River |
| C61-6 | Astyanax mexicanus | Astyanax mexicanus | Cuatro Ciénegas | Bravo River |
| C61-7 | Astyanax mexicanus | Astyanax mexicanus | Cuatro Ciénegas | Bravo River |
| C61-8 | Astyanax mexicanus | Astyanax mexicanus | Cuatro Ciénegas | Bravo River |
| C61-9 | Astyanax mexicanus | Astyanax mexicanus | Cuatro Ciénegas | Bravo River |
| C61-10 | Astyanax mexicanus | Astyanax mexicanus | Cuatro Ciénegas | Bravo River |
| C61-11 | Astyanax mexicanus | Astyanax mexicanus | Cuatro Ciénegas | Bravo River |
| C61-12 | Astyanax mexicanus | Astyanax mexicanus | Cuatro Ciénegas | Bravo River |
| C61-13 | Astyanax mexicanus | Astyanax mexicanus | Cuatro Ciénegas | Bravo River |
| C61-14 | Astyanax mexicanus | Astyanax mexicanus | Cuatro Ciénegas | Bravo River |
| C61-15 | Astyanax mexicanus | Astyanax mexicanus | Cuatro Ciénegas | Bravo River |
| C61-16 | Astyanax mexicanus | Astyanax mexicanus | Cuatro Ciénegas | Bravo River |
| C61-17 | Astyanax mexicanus | Astyanax mexicanus | Cuatro Ciénegas | Bravo River |
| C61-18 | Astyanax mexicanus | Astyanax mexicanus | Cuatro Ciénegas | Bravo River |
| C61-19 | Astyanax mexicanus | Astyanax mexicanus | Cuatro Ciénegas | Bravo River |
| C61-20 | Astyanax mexicanus | Astyanax mexicanus | Cuatro Ciénegas | Bravo River |
| C61-21 | Astyanax mexicanus | Astyanax mexicanus | Cuatro Ciénegas | Bravo River |
| C61-22 | Astyanax mexicanus | Astyanax mexicanus | Cuatro Ciénegas | Bravo River |
| C61-23 | Astyanax mexicanus | Astyanax mexicanus | Cuatro Ciénegas | Bravo River |
| C61-24 | Astyanax mexicanus | Astyanax mexicanus | Cuatro Ciénegas | Bravo River |
| C61-25 | Astyanax mexicanus | Astyanax mexicanus | Cuatro Ciénegas | Bravo River |
| C61-26 | Astyanax mexicanus | Astyanax mexicanus | Cuatro Ciénegas | Bravo River |
| C61-27 | Astyanax mexicanus | Astyanax mexicanus | San Fernando | San Fernando |
| C61-28 | Astyanax mexicanus | Astyanax mexicanus | San Fernando | San Fernando |
| C61-29 | Astyanax mexicanus | Astyanax mexicanus | San Fernando | San Fernando |
| C61-30 | Astyanax mexicanus | Astyanax mexicanus | San Fernando | San Fernando |
| C61-31 | Astyanax mexicanus | Astyanax mexicanus | San Fernando | San Fernando |
| C61-32 | Astyanax mexicanus | Astyanax mexicanus | San Fernando | San Fernando |
| C61-33 | Astyanax mexicanus | Astyanax mexicanus | San Fernando | San Fernando |
| C61-34 | Astyanax mexicanus | Astyanax mexicanus | San Fernando | San Fernando |
| C61-35 | Astyanax mexicanus | Astyanax mexicanus | San Fernando | San Fernando |
| C61-36 | Astyanax mexicanus | Astyanax mexicanus | San Fernando | San Fernando |
| C61-37 | Astyanax mexicanus | Astyanax mexicanus | San Fernando | San Fernando |
| C61-38 | Astyanax mexicanus | Astyanax mexicanus | San Fernando | San Fernando |
| C61-39 | Astyanax mexicanus | Astyanax mexicanus | San Fernando | San Fernando |
| C61-40 | Astyanax mexicanus | Astyanax mexicanus | San Fernando | San Fernando |
| C61-41 | Astyanax mexicanus | Astyanax mexicanus | San Fernando | San Fernando |
| C61-42 | Astyanax mexicanus | Astyanax mexicanus | San Fernando | San Fernando |
| C61-43 | Astyanax mexicanus | Astyanax mexicanus | San Fernando | San Fernando |
| C61-44 | Astyanax mexicanus | Astyanax mexicanus | San Fernando | San Fernando |
| C61-45 | Astyanax mexicanus | Astyanax mexicanus | San Fernando | San Fernando |
| C61-46 | Astyanax mexicanus | Astyanax mexicanus | San Fernando | San Fernando |
| C61-47 | Astyanax mexicanus | Astyanax mexicanus | San Fernando | San Fernando |
| C61-48 | Astyanax mexicanus | Astyanax mexicanus | San Fernando | San Fernando |
| C61-49 | Astyanax mexicanus | Astyanax mexicanus | San Fernando | San Fernando |
| C61-50 | Astyanax mexicanus | Astyanax mexicanus | San Fernando | San Fernando |
| C61-51 | Astyanax mexicanus | Astyanax mexicanus | San Fernando | San Fernando |
| C61-52 | Astyanax mexicanus | Astyanax mexicanus | San Fernando | San Fernando |
| C61-53 | Astyanax mexicanus | Astyanax mexicanus | San Fernando | San Fernando |
| C61-54 | Astyanax mexicanus | Astyanax mexicanus | San Fernando | San Fernando |
| C61-55 | Astyanax mexicanus | Astyanax mexicanus | San Fernando | San Fernando |
| C61-56 | Astyanax mexicanus | Astyanax mexicanus | San Fernando | San Fernando |
| C61-57 | Astyanax mexicanus | Astyanax mexicanus | Garza Valdez | Soto la Marina |
| C61-58 | Astyanax mexicanus | Astyanax mexicanus | Garza Valdez | Soto la Marina |
| C61-59 | Astyanax mexicanus | Astyanax mexicanus | Garza Valdez | Soto la Marina |
| C61-60 | Astyanax mexicanus | Astyanax mexicanus | Garza Valdez | Soto la Marina |
| C61-61 | Astyanax mexicanus | Astyanax mexicanus | Garza Valdez | Soto la Marina |
| C61-62 | Astyanax mexicanus | Astyanax mexicanus | Garza Valdez | Soto la Marina |
| C61-63 | Astyanax mexicanus | Astyanax mexicanus | Garza Valdez | Soto la Marina |
| C61-64 | Astyanax mexicanus | Astyanax mexicanus | Garza Valdez | Soto la Marina |
| C61-65 | Astyanax mexicanus | Astyanax mexicanus | Garza Valdez | Soto la Marina |
| C61-66 | Astyanax mexicanus | Astyanax mexicanus | Garza Valdez | Soto la Marina |
| C61-67 | Astyanax mexicanus | Astyanax mexicanus | Garza Valdez | Soto la Marina |
| C61-68 | Astyanax mexicanus | Astyanax mexicanus | Garza Valdez | Soto la Marina |
| C61-69 | Astyanax mexicanus | Astyanax mexicanus | Garza Valdez | Soto la Marina |
| C61-70 | Astyanax mexicanus | Astyanax mexicanus | Garza Valdez | Soto la Marina |
| C61-71 | Astyanax mexicanus | Astyanax mexicanus | Garza Valdez | Soto la Marina |
| C61-72 | Astyanax mexicanus | Astyanax mexicanus | Garza Valdez | Soto la Marina |
| C61-73 | Astyanax mexicanus | Astyanax mexicanus | Garza Valdez | Soto la Marina |
| C61-74 | Astyanax mexicanus | Astyanax mexicanus | Garza Valdez | Soto la Marina |
| C61-75 | Astyanax mexicanus | Astyanax mexicanus | Garza Valdez | Soto la Marina |
| C61-76 | Astyanax mexicanus | Astyanax mexicanus | Garza Valdez | Soto la Marina |
| C61-77 | Astyanax mexicanus | Astyanax mexicanus | Garza Valdez | Soto la Marina |
| C61-78 | Astyanax mexicanus | Astyanax mexicanus | Garza Valdez | Soto la Marina |
| C61-79 | Astyanax mexicanus | Astyanax mexicanus | Garza Valdez | Soto la Marina |
| C61-80 | Astyanax mexicanus | Astyanax mexicanus | Garza Valdez | Soto la Marina |
| C61-81 | Astyanax mexicanus | Astyanax mexicanus | Troncones | Soto la Marina |
| C61-82 | Astyanax mexicanus | Astyanax mexicanus | Troncones | Soto la Marina |
| C61-83 | Astyanax mexicanus | Astyanax mexicanus | Troncones | Soto la Marina |
| C61-84 | Astyanax mexicanus | Astyanax mexicanus | Troncones | Soto la Marina |
| C61-85 | Astyanax mexicanus | Astyanax mexicanus | Troncones | Soto la Marina |
| C61-86 | Astyanax mexicanus | Astyanax mexicanus | Troncones | Soto la Marina |
| C61-87 | Astyanax mexicanus | Astyanax mexicanus | Troncones | Soto la Marina |
| C61-88 | Astyanax mexicanus | Astyanax mexicanus | Troncones | Soto la Marina |
| C61-89 | Astyanax mexicanus | Astyanax mexicanus | Troncones | Soto la Marina |
| C61-90 | Astyanax mexicanus | Astyanax mexicanus | Troncones | Soto la Marina |
| C61-91 | Astyanax mexicanus | Astyanax mexicanus | Troncones | Soto la Marina |
| C61-92 | Astyanax mexicanus | Astyanax mexicanus | Troncones | Soto la Marina |
| C61-93 | Astyanax mexicanus | Astyanax mexicanus | Troncones | Soto la Marina |
| C61-94 | Astyanax mexicanus | Astyanax mexicanus | Troncones | Soto la Marina |
| C61-95 | Astyanax mexicanus | Astyanax mexicanus | Troncones | Soto la Marina |
| C61-96 | Astyanax mexicanus | Astyanax mexicanus | Troncones | Soto la Marina |
| C61-97 | Astyanax mexicanus | Astyanax mexicanus | Troncones | Soto la Marina |
| C61-98 | Astyanax mexicanus | Astyanax mexicanus | Troncones | Soto la Marina |
| C61-99 | Astyanax mexicanus | Astyanax mexicanus | Troncones | Soto la Marina |
| C61-100 | Astyanax mexicanus | Astyanax mexicanus | Troncones | Soto la Marina |
| C62-101 | Astyanax mexicanus | Astyanax mexicanus | Troncones | Soto la Marina |
| C62-102 | Astyanax mexicanus | Astyanax mexicanus | Troncones | Soto la Marina |
| C62-103 | Astyanax mexicanus | Astyanax mexicanus | Troncones | Soto la Marina |
| C62-104 | Astyanax mexicanus | Astyanax mexicanus | Troncones | Soto la Marina |
| C62-105 | Astyanax mexicanus | Astyanax mexicanus | Troncones | Soto la Marina |
| C62-106 | Astyanax mexicanus | Astyanax mexicanus | Troncones | Soto la Marina |
| C62-107 | Astyanax mexicanus | Astyanax mexicanus | Troncones | Soto la Marina |
| C62-108 | Astyanax mexicanus | Astyanax mexicanus | Troncones | Soto la Marina |
| C62-109 | Astyanax mexicanus | Astyanax mexicanus | Troncones | Soto la Marina |
| C62-110 | Astyanax mexicanus | Astyanax mexicanus | Troncones | Soto la Marina |
| C62-111 | Astyanax mexicanus | Astyanax mexicanus | Troncones | Soto la Marina |
| C62-112 | Astyanax mexicanus | Astyanax mexicanus | Troncones | Soto la Marina |
| C62-113 | Astyanax mexicanus | Astyanax mexicanus | Troncones | Soto la Marina |
| C62-114 | Astyanax mexicanus | Astyanax mexicanus | Troncones | Soto la Marina |
| C62-115 | Astyanax mexicanus | Astyanax mexicanus | Troncones | Soto la Marina |
| C62-116 | Astyanax mexicanus | Astyanax mexicanus | Troncones | Soto la Marina |
| C62-117 | Astyanax mexicanus | Astyanax mexicanus | Troncones | Soto la Marina |
| C62-118 | Astyanax mexicanus | Astyanax mexicanus | Troncones | Soto la Marina |
| C62-119 | Astyanax mexicanus | Astyanax mexicanus | Troncones | Soto la Marina |
| C62-120 | Astyanax mexicanus | Astyanax mexicanus | Troncones | Soto la Marina |
| C62-121 | Astyanax mexicanus | Astyanax mexicanus | Arroyo Lagartos | Pánuco |
| C62-122 | Astyanax mexicanus | Astyanax mexicanus | Arroyo Lagartos | Pánuco |
| C62-123 | Astyanax mexicanus | Astyanax mexicanus | Arroyo Lagartos | Pánuco |
| C62-124 | Astyanax mexicanus | Astyanax mexicanus | Arroyo Lagartos | Pánuco |
| C62-125 | Astyanax mexicanus | Astyanax mexicanus | Arroyo Lagartos | Pánuco |
| C62-126 | Astyanax mexicanus | Astyanax mexicanus | Arroyo Lagartos | Pánuco |
| C62-127 | Astyanax mexicanus | Astyanax mexicanus | Arroyo Lagartos | Pánuco |
| C62-128 | Astyanax mexicanus | Astyanax mexicanus | Arroyo Lagartos | Pánuco |
| C62-129 | Astyanax mexicanus | Astyanax mexicanus | Arroyo Lagartos | Pánuco |
| C62-130 | Astyanax mexicanus | Astyanax mexicanus | Arroyo Lagartos | Pánuco |
| C62-131 | Astyanax mexicanus | Astyanax mexicanus | Arroyo Lagartos | Pánuco |
| C62-132 | Astyanax mexicanus | Astyanax mexicanus | Arroyo Lagartos | Pánuco |
| C62-133 | Astyanax mexicanus | Astyanax mexicanus | Arroyo Lagartos | Pánuco |
| C62-134 | Astyanax mexicanus | Astyanax mexicanus | Arroyo Lagartos | Pánuco |
| C62-135 | Astyanax mexicanus | Astyanax mexicanus | Arroyo Lagartos | Pánuco |
| C62-136 | Astyanax mexicanus | Astyanax mexicanus | Arroyo Lagartos | Pánuco |
| C62-137 | Astyanax mexicanus | Astyanax mexicanus | Arroyo Lagartos | Pánuco |
| C62-138 | Astyanax mexicanus | Astyanax mexicanus | Arroyo Lagartos | Pánuco |
| C62-139 | Astyanax mexicanus | Astyanax mexicanus | Arroyo Lagartos | Pánuco |
| C62-140 | Astyanax mexicanus | Astyanax mexicanus | Arroyo Lagartos | Pánuco |
| C62-141 | Astyanax mexicanus | Astyanax mexicanus | Arroyo Lagartos | Pánuco |
| C62-142 | Astyanax mexicanus | Astyanax mexicanus | Arroyo Lagartos | Pánuco |
| C62-143 | Astyanax mexicanus | Astyanax mexicanus | Arroyo Lagartos | Pánuco |
| C62-144 | Astyanax mexicanus | Astyanax mexicanus | Arroyo Lagartos | Pánuco |
| C62-145 | Astyanax mexicanus | Astyanax mexicanus | Arroyo Lagartos | Pánuco |
| C62-146 | Astyanax mexicanus | Astyanax mexicanus | Arroyo Lagartos | Pánuco |
| C62-147 | Astyanax mexicanus | Astyanax mexicanus | Arroyo Lagartos | Pánuco |
| C62-148 | Astyanax mexicanus | Astyanax mexicanus | Arroyo Lagartos | Pánuco |
| C62-149 | Astyanax mexicanus | Astyanax mexicanus | Arroyo Lagartos | Pánuco |
| C62-150 | Astyanax mexicanus | Astyanax mexicanus | Arroyo Lagartos | Pánuco |
| C62-151 | Astyanax mexicanus | Astyanax mexicanus | Arroyo Lagartos | Pánuco |
| C62-152 | Astyanax mexicanus | Astyanax mexicanus | Arroyo Lagartos | Pánuco |
| C62-153 | Astyanax mexicanus | Astyanax mexicanus | Arroyo Lagartos | Pánuco |
| C62-154 | Astyanax mexicanus | Astyanax mexicanus | Arroyo Lagartos | Pánuco |
| C62-155 | Astyanax mexicanus | Astyanax mexicanus | Arroyo Lagartos | Pánuco |
| C62-156 | Astyanax mexicanus | Astyanax mexicanus | Arroyo Lagartos | Pánuco |
| C62-157 | Astyanax mexicanus | Astyanax mexicanus | Molino | Pánuco |
| C62-158 | Astyanax mexicanus | Astyanax mexicanus | Molino | Pánuco |
| C62-159 | Astyanax mexicanus | Astyanax mexicanus | Molino | Pánuco |
| C62-160 | Astyanax mexicanus | Astyanax mexicanus | Molino | Pánuco |
| C62-161 | Astyanax mexicanus | Astyanax mexicanus | Molino | Pánuco |
| C62-162 | Astyanax mexicanus | Astyanax mexicanus | Molino | Pánuco |
| C62-163 | Astyanax mexicanus | Astyanax mexicanus | Molino | Pánuco |
| C62-164 | Astyanax mexicanus | Astyanax mexicanus | Molino | Pánuco |
| C62-165 | Astyanax mexicanus | Astyanax mexicanus | Molino | Pánuco |
| C62-166 | Astyanax mexicanus | Astyanax mexicanus | Molino | Pánuco |
| C62-167 | Astyanax mexicanus | Astyanax mexicanus | Molino | Pánuco |
| C62-168 | Astyanax mexicanus | Astyanax mexicanus | Molino | Pánuco |
| C62-169 | Astyanax mexicanus | Astyanax mexicanus | Molino | Pánuco |
| C62-170 | Astyanax mexicanus | Astyanax mexicanus | Molino | Pánuco |
| C62-171 | Astyanax mexicanus | Astyanax mexicanus | Molino | Pánuco |
| C62-172 | Astyanax mexicanus | Astyanax mexicanus | Pachón | Pánuco |
| C62-173 | Astyanax mexicanus | Astyanax mexicanus | Pachón | Pánuco |
| C62-174 | Astyanax mexicanus | Astyanax mexicanus | Pachón | Pánuco |
| C62-175 | Astyanax mexicanus | Astyanax mexicanus | Pachón | Pánuco |
| C62-176 | Astyanax mexicanus | Astyanax mexicanus | Pachón | Pánuco |
| C62-177 | Astyanax mexicanus | Astyanax mexicanus | Pachón | Pánuco |
| C62-178 | Astyanax mexicanus | Astyanax mexicanus | Pachón | Pánuco |
| C62-179 | Astyanax mexicanus | Astyanax mexicanus | Pachón | Pánuco |
| C62-180 | Astyanax mexicanus | Astyanax mexicanus | Pachón | Pánuco |
| C62-181 | Astyanax mexicanus | Astyanax mexicanus | Pachón | Pánuco |
| C62-182 | Astyanax mexicanus | Astyanax mexicanus | Pachón | Pánuco |
| C62-183 | Astyanax mexicanus | Astyanax mexicanus | Pachón | Pánuco |
| C62-184 | Astyanax mexicanus | Astyanax mexicanus | Pachón | Pánuco |
| C62-185 | Astyanax mexicanus | Astyanax mexicanus | Pachón | Pánuco |
| C62-186 | Astyanax mexicanus | Astyanax mexicanus | Pachón | Pánuco |
| C62-187 | Astyanax mexicanus | Astyanax mexicanus | Pachón | Pánuco |
| C62-188 | Astyanax mexicanus | Astyanax mexicanus | Pachón | Pánuco |
| C62-189 | Astyanax mexicanus | Astyanax mexicanus | Pachón | Pánuco |
| C62-190 | Astyanax mexicanus | Astyanax mexicanus | Pachón | Pánuco |
| C62-191 | Astyanax mexicanus | Astyanax mexicanus | Pachón | Pánuco |
| C62-192 | Astyanax mexicanus | Astyanax mexicanus | Pachón | Pánuco |
| C62-193 | Astyanax mexicanus | Astyanax mexicanus | Pachón | Pánuco |
| C62-194 | Astyanax mexicanus | Astyanax mexicanus | Pachón | Pánuco |
| C62-195 | Astyanax mexicanus | Astyanax mexicanus | Pachón | Pánuco |
| C62-196 | Astyanax mexicanus | Astyanax mexicanus | Pachón | Pánuco |
| C62-197 | Astyanax mexicanus | Astyanax mexicanus | Pachón | Pánuco |
| C62-198 | Astyanax mexicanus | Astyanax mexicanus | Pachón | Pánuco |
| C62-199 | Astyanax mexicanus | Astyanax mexicanus | Pachón | Pánuco |
| C62-200 | Astyanax mexicanus | Astyanax mexicanus | Pachón | Pánuco |
| C63-201 | Astyanax mexicanus | Astyanax mexicanus | Pachón | Pánuco |
| C63-202 | Astyanax mexicanus | Astyanax mexicanus | Pachón | Pánuco |
| C63-203 | Astyanax mexicanus | Astyanax mexicanus | Pachón | Pánuco |
| C63-204 | Astyanax mexicanus | Astyanax mexicanus | Pachón | Pánuco |
| C63-205 | Astyanax mexicanus | Astyanax mexicanus | Pachón | Pánuco |
| C63-206 | Astyanax mexicanus | Astyanax mexicanus | Pachón | Pánuco |
| C63-207 | Astyanax mexicanus | Astyanax mexicanus | Pachón | Pánuco |
| C63-208 | Astyanax mexicanus | Astyanax mexicanus | Pachón | Pánuco |
| C63-209 | Astyanax mexicanus | Astyanax mexicanus | Pachón | Pánuco |
| C63-210 | Astyanax mexicanus | Astyanax mexicanus | Pachón | Pánuco |
| C63-211 | Astyanax mexicanus | Astyanax mexicanus | Pachón | Pánuco |
| C63-212 | Astyanax mexicanus | Astyanax mexicanus | Pachón | Pánuco |
| C63-213 | Astyanax mexicanus | Astyanax mexicanus | Pachón | Pánuco |
| C63-214 | Astyanax mexicanus | Astyanax mexicanus | Pachón | Pánuco |
| C63-215 | Astyanax mexicanus | Astyanax mexicanus | Pachón | Pánuco |
| C63-216 | Astyanax mexicanus | Astyanax hubbsi | Sabinos | Pánuco |
| C63-217 | Astyanax mexicanus | Astyanax hubbsi | Sabinos | Pánuco |
| C63-218 | Astyanax mexicanus | Astyanax hubbsi | Sabinos | Pánuco |
| C63-219 | Astyanax mexicanus | Astyanax hubbsi | Sabinos | Pánuco |
| C63-220 | Astyanax mexicanus | Astyanax hubbsi | Sabinos | Pánuco |
| C63-221 | Astyanax mexicanus | Astyanax hubbsi | Sabinos | Pánuco |
| C63-222 | Astyanax mexicanus | Astyanax hubbsi | Sabinos | Pánuco |
| C63-223 | Astyanax mexicanus | Astyanax hubbsi | Sabinos | Pánuco |
| C63-224 | Astyanax mexicanus | Astyanax hubbsi | Sabinos | Pánuco |
| C63-225 | Astyanax mexicanus | Astyanax hubbsi | Sabinos | Pánuco |
| C63-226 | Astyanax mexicanus | Astyanax hubbsi | Sabinos | Pánuco |
| C63-227 | Astyanax mexicanus | Astyanax hubbsi | Sabinos | Pánuco |
| C63-228 | Astyanax mexicanus | Astyanax hubbsi | Sabinos | Pánuco |
| C63-229 | Astyanax mexicanus | Astyanax hubbsi | Sabinos | Pánuco |
| C63-230 | Astyanax mexicanus | Astyanax hubbsi | Sabinos | Pánuco |
| C63-231 | Astyanax mexicanus | Astyanax hubbsi | Sabinos | Pánuco |
| C63-232 | Astyanax mexicanus | Astyanax hubbsi | Sabinos | Pánuco |
| C63-233 | Astyanax mexicanus | Astyanax hubbsi | Sabinos | Pánuco |
| C63-234 | Astyanax mexicanus | Astyanax hubbsi | Sabinos | Pánuco |
| C63-235 | Astyanax mexicanus | Astyanax hubbsi | Sabinos | Pánuco |
| C63-236 | Astyanax mexicanus | Astyanax hubbsi | Sabinos | Pánuco |
| C63-237 | Astyanax mexicanus | Astyanax hubbsi | Sabinos | Pánuco |
| C63-238 | Astyanax mexicanus | Astyanax hubbsi | Sabinos | Pánuco |
| C63-239 | Astyanax mexicanus | Astyanax hubbsi | Tinajas | Pánuco |
| C63-240 | Astyanax mexicanus | Astyanax hubbsi | Tinajas | Pánuco |
| C63-241 | Astyanax mexicanus | Astyanax hubbsi | Tinajas | Pánuco |
| C63-242 | Astyanax mexicanus | Astyanax hubbsi | Tinajas | Pánuco |
| C63-243 | Astyanax mexicanus | Astyanax hubbsi | Tinajas | Pánuco |
| C63-244 | Astyanax mexicanus | Astyanax hubbsi | Tinajas | Pánuco |
| C63-245 | Astyanax mexicanus | Astyanax hubbsi | Tinajas | Pánuco |
| C63-246 | Astyanax mexicanus | Astyanax hubbsi | Tinajas | Pánuco |
| C63-247 | Astyanax mexicanus | Astyanax hubbsi | Tinajas | Pánuco |
| C63-248 | Astyanax mexicanus | Astyanax hubbsi | Tinajas | Pánuco |
| C63-249 | Astyanax mexicanus | Astyanax hubbsi | Tinajas | Pánuco |
| C63-250 | Astyanax mexicanus | Astyanax hubbsi | Tinajas | Pánuco |
| C63-251 | Astyanax mexicanus | Astyanax mexicanus | La Cañada | Pánuco |
| C63-252 | Astyanax mexicanus | Astyanax mexicanus | La Cañada | Pánuco |
| C63-253 | Astyanax mexicanus | Astyanax mexicanus | La Cañada | Pánuco |
| C63-254 | Astyanax mexicanus | Astyanax mexicanus | La Cañada | Pánuco |
| C63-255 | Astyanax mexicanus | Astyanax mexicanus | La Cañada | Pánuco |
| C63-256 | Astyanax mexicanus | Astyanax mexicanus | La Cañada | Pánuco |
| C63-257 | Astyanax mexicanus | Astyanax mexicanus | La Cañada | Pánuco |
| C63-258 | Astyanax mexicanus | Astyanax mexicanus | La Cañada | Pánuco |
| C63-259 | Astyanax mexicanus | Astyanax mexicanus | La Cañada | Pánuco |
| C63-260 | Astyanax mexicanus | Astyanax mexicanus | La Cañada | Pánuco |
| C63-261 | Astyanax mexicanus | Astyanax mexicanus | La Cañada | Pánuco |
| C63-262 | Astyanax mexicanus | Astyanax mexicanus | La Cañada | Pánuco |
| C63-263 | Astyanax mexicanus | Astyanax mexicanus | La Cañada | Pánuco |
| C63-264 | Astyanax mexicanus | Astyanax mexicanus | La Cañada | Pánuco |
| C63-265 | Astyanax mexicanus | Astyanax mexicanus | La Cañada | Pánuco |
| C63-266 | Astyanax mexicanus | Astyanax mexicanus | La Cañada | Pánuco |
| C63-267 | Astyanax mexicanus | Astyanax mexicanus | La Cañada | Pánuco |
| C63-268 | Astyanax mexicanus | Astyanax mexicanus | La Cañada | Pánuco |
| C63-269 | Astyanax mexicanus | Astyanax mexicanus | La Cañada | Pánuco |
| C63-270 | Astyanax mexicanus | Astyanax mexicanus | La Cañada | Pánuco |
| C63-271 | Astyanax mexicanus | Astyanax mexicanus | La Cañada | Pánuco |
| C63-272 | Astyanax mexicanus | Astyanax mexicanus | La Cañada | Pánuco |
| C63-273 | Astyanax mexicanus | Astyanax mexicanus | La Cañada | Pánuco |
| C63-274 | Astyanax mexicanus | Astyanax mexicanus | La Cañada | Pánuco |
| C63-275 | Astyanax mexicanus | Astyanax mexicanus | La Cañada | Pánuco |
| C63-276 | Astyanax mexicanus | Astyanax mexicanus | La Cañada | Pánuco |
| C63-277 | Astyanax mexicanus | Astyanax mexicanus | Huichihuayan | Pánuco |
| C63-278 | Astyanax mexicanus | Astyanax mexicanus | Huichihuayan | Pánuco |
| C63-279 | Astyanax mexicanus | Astyanax mexicanus | Huichihuayan | Pánuco |
| C63-280 | Astyanax mexicanus | Astyanax mexicanus | Huichihuayan | Pánuco |
| C63-281 | Astyanax mexicanus | Astyanax mexicanus | Huichihuayan | Pánuco |
| C63-282 | Astyanax mexicanus | Astyanax mexicanus | Huichihuayan | Pánuco |
| C63-283 | Astyanax mexicanus | Astyanax mexicanus | Huichihuayan | Pánuco |
| C63-284 | Astyanax mexicanus | Astyanax mexicanus | Huichihuayan | Pánuco |
| C63-285 | Astyanax mexicanus | Astyanax mexicanus | Huichihuayan | Pánuco |
| C63-286 | Astyanax mexicanus | Astyanax mexicanus | Huichihuayan | Pánuco |
| C63-287 | Astyanax mexicanus | Astyanax mexicanus | Huichihuayan | Pánuco |
| C63-288 | Astyanax mexicanus | Astyanax mexicanus | Huichihuayan | Pánuco |
| C63-289 | Astyanax mexicanus | Astyanax mexicanus | Huichihuayan | Pánuco |
| C63-290 | Astyanax mexicanus | Astyanax mexicanus | Huichihuayan | Pánuco |
| C63-291 | Astyanax mexicanus | Astyanax mexicanus | Huichihuayan | Pánuco |
| C63-292 | Astyanax mexicanus | Astyanax mexicanus | Huichihuayan | Pánuco |
| C63-293 | Astyanax mexicanus | Astyanax mexicanus | Huichihuayan | Pánuco |
| C63-294 | Astyanax mexicanus | Astyanax mexicanus | Huichihuayan | Pánuco |
| C63-295 | Astyanax mexicanus | Astyanax mexicanus | Huichihuayan | Pánuco |
| C63-296 | Astyanax mexicanus | Astyanax aeneus | Zapotal | Tuxpan |
| C63-297 | Astyanax mexicanus | Astyanax aeneus | Zapotal | Tuxpan |
| C63-298 | Astyanax mexicanus | Astyanax aeneus | Zapotal | Tuxpan |
| C63-299 | Astyanax mexicanus | Astyanax aeneus | Zapotal | Tuxpan |
| C64-300 | Astyanax mexicanus | Astyanax aeneus | Zapotal | Tuxpan |
| C64-301 | Astyanax mexicanus | Astyanax aeneus | Zapotal | Tuxpan |
| C64-302 | Astyanax mexicanus | Astyanax aeneus | Zapotal | Tuxpan |
| C64-303 | Astyanax mexicanus | Astyanax aeneus | Zapotal | Tuxpan |
| C64-304 | Astyanax mexicanus | Astyanax aeneus | Zapotal | Tuxpan |
| C64-305 | Astyanax mexicanus | Astyanax aeneus | Zapotal | Tuxpan |
| C64-306 | Astyanax mexicanus | Astyanax aeneus | Zapotal | Tuxpan |
| C64-307 | Astyanax mexicanus | Astyanax aeneus | Zapotal | Tuxpan |
| C64-308 | Astyanax mexicanus | Astyanax aeneus | Zapotal | Tuxpan |
| C64-309 | Astyanax mexicanus | Astyanax aeneus | Zapotal | Tuxpan |
| C64-310 | Astyanax mexicanus | Astyanax aeneus | Zapotal | Tuxpan |
| C64-311 | Astyanax mexicanus | Astyanax aeneus | Zapotal | Tuxpan |
| C64-312 | Astyanax mexicanus | Astyanax aeneus | Zapotal | Tuxpan |
| C64-313 | Astyanax mexicanus | Astyanax aeneus | Zapotal | Tuxpan |
| C64-314 | Astyanax mexicanus | Astyanax aeneus | Zapotal | Tuxpan |
| C64-315 | Astyanax mexicanus | Astyanax aeneus | Zapotal | Tuxpan |
| C64-316 | Astyanax mexicanus | Astyanax aeneus | Zapotal | Tuxpan |
| C64-317 | Astyanax mexicanus | Astyanax aeneus | Zapotal | Tuxpan |
| C64-318 | Astyanax mexicanus | Astyanax aeneus | Zapotal | Tuxpan |
| C64-319 | Astyanax mexicanus | Astyanax aeneus | Zapotal | Tuxpan |
| C64-320 | Astyanax mexicanus | Astyanax aeneus | Zapotal | Tuxpan |
| C64-321 | Astyanax mexicanus | Astyanax aeneus | Zapotal | Tuxpan |
| C64-322 | Astyanax mexicanus | Astyanax aeneus | Zapotal | Tuxpan |
| C64-323 | Astyanax mexicanus | Astyanax aeneus | Zapotal | Tuxpan |
| C64-324 | Astyanax mexicanus | Astyanax aeneus | Zapotal | Tuxpan |
| C64-325 | Astyanax mexicanus | Astyanax aeneus | Zapotal | Tuxpan |
| C64-326 | Astyanax mexicanus | Astyanax aeneus | Catemaco | Papaloapan |
| C64-327 | Astyanax mexicanus | Astyanax aeneus | Catemaco | Papaloapan |
| C64-328 | Astyanax mexicanus | Astyanax aeneus | Catemaco | Papaloapan |
| C64-329 | Astyanax mexicanus | Astyanax aeneus | Catemaco | Papaloapan |
| C64-330 | Astyanax mexicanus | Astyanax aeneus | Catemaco | Papaloapan |
| C64-331 | Astyanax mexicanus | Astyanax aeneus | Catemaco | Papaloapan |
| C64-332 | Astyanax mexicanus | Astyanax aeneus | Catemaco | Papaloapan |
| C64-333 | Astyanax mexicanus | Astyanax aeneus | Catemaco | Papaloapan |
| C64-334 | Astyanax mexicanus | Astyanax aeneus | Catemaco | Papaloapan |
| C64-335 | Astyanax mexicanus | Astyanax aeneus | Catemaco | Papaloapan |
| C64-336 | Astyanax mexicanus | Astyanax aeneus | Catemaco | Papaloapan |
| C64-337 | Astyanax mexicanus | Astyanax aeneus | Catemaco | Papaloapan |
| C64-338 | Astyanax mexicanus | Astyanax aeneus | Catemaco | Papaloapan |
| C64-339 | Astyanax mexicanus | Astyanax aeneus | Catemaco | Papaloapan |
| C64-340 | Astyanax mexicanus | Astyanax aeneus | Catemaco | Papaloapan |
| C64-341 | Astyanax mexicanus | Astyanax aeneus | Catemaco | Papaloapan |
| C64-342 | Astyanax mexicanus | Astyanax aeneus | Catemaco | Papaloapan |
| C64-343 | Astyanax mexicanus | Astyanax aeneus | Catemaco | Papaloapan |
| C64-344 | Astyanax mexicanus | Astyanax aeneus | Catemaco | Papaloapan |
| C64-345 | Astyanax mexicanus | Astyanax aeneus | Catemaco | Papaloapan |
| C64-346 | Astyanax mexicanus | Astyanax aeneus | Catemaco | Papaloapan |
| C64-347 | Astyanax mexicanus | Astyanax aeneus | Catemaco | Papaloapan |
| C64-348 | Astyanax mexicanus | Astyanax aeneus | Catemaco | Papaloapan |
| C64-349 | Astyanax mexicanus | Astyanax aeneus | Catemaco | Papaloapan |
| C64-350 | Astyanax mexicanus | Astyanax aeneus | Catemaco | Papaloapan |
| C64-351 | Astyanax mexicanus | Astyanax aeneus | Catemaco | Papaloapan |
| C64-352 | Astyanax mexicanus | Astyanax aeneus | Teapa | Grijalva-Usumacinta |
| C64-353 | Astyanax mexicanus | Astyanax aeneus | Teapa | Grijalva-Usumacinta |
| C64-354 | Astyanax mexicanus | Astyanax aeneus | Teapa | Grijalva-Usumacinta |
| C64-355 | Astyanax mexicanus | Astyanax aeneus | Teapa | Grijalva-Usumacinta |
| C64-356 | Astyanax mexicanus | Astyanax aeneus | Teapa | Grijalva-Usumacinta |
| C64-357 | Astyanax mexicanus | Astyanax aeneus | Teapa | Grijalva-Usumacinta |
| C64-358 | Astyanax mexicanus | Astyanax aeneus | Teapa | Grijalva-Usumacinta |
| C64-359 | Astyanax mexicanus | Astyanax aeneus | Teapa | Grijalva-Usumacinta |
| C64-360 | Astyanax mexicanus | Astyanax aeneus | Teapa | Grijalva-Usumacinta |
| C64-361 | Astyanax mexicanus | Astyanax aeneus | Teapa | Grijalva-Usumacinta |
| C64-362 | Astyanax mexicanus | Astyanax aeneus | Teapa | Grijalva-Usumacinta |
| C64-363 | Astyanax mexicanus | Astyanax aeneus | Teapa | Grijalva-Usumacinta |
| C64-364 | Astyanax mexicanus | Astyanax aeneus | Teapa | Grijalva-Usumacinta |
| C64-365 | Astyanax mexicanus | Astyanax aeneus | Teapa | Grijalva-Usumacinta |
| C64-366 | Astyanax mexicanus | Astyanax aeneus | Teapa | Grijalva-Usumacinta |
| C64-367 | Astyanax mexicanus | Astyanax aeneus | Teapa | Grijalva-Usumacinta |
| C64-368 | Astyanax mexicanus | Astyanax aeneus | Teapa | Grijalva-Usumacinta |
| C64-369 | Astyanax mexicanus | Astyanax aeneus | Teapa | Grijalva-Usumacinta |
| C64-370 | Astyanax mexicanus | Astyanax aeneus | Teapa | Grijalva-Usumacinta |
| C64-371 | Astyanax mexicanus | Astyanax aeneus | Teapa | Grijalva-Usumacinta |
| C64-372 | Astyanax mexicanus | Astyanax aeneus | Teapa | Grijalva-Usumacinta |
| C64-373 | Astyanax mexicanus | Astyanax aeneus | Teapa | Grijalva-Usumacinta |
| C64-374 | Astyanax mexicanus | Astyanax aeneus | Teapa | Grijalva-Usumacinta |
| C64-375 | Astyanax mexicanus | Astyanax aeneus | Teapa | Grijalva-Usumacinta |
| C64-376 | Astyanax mexicanus | Astyanax aeneus | Teapa | Grijalva-Usumacinta |
| C64-377 | Astyanax mexicanus | Astyanax aeneus | Teapa | Grijalva-Usumacinta |
| C64-378 | Astyanax mexicanus | Astyanax aeneus | Teapa | Grijalva-Usumacinta |
| C64-379 | Astyanax mexicanus | Astyanax aeneus | Teapa | Grijalva-Usumacinta |
| C64-380 | Astyanax mexicanus | Astyanax aeneus | Teapa | Grijalva-Usumacinta |
| C64-381 | Astyanax mexicanus | Astyanax aeneus | Teapa | Grijalva-Usumacinta |
| C64-382 | Astyanax mexicanus | Astyanax aeneus | Teapa | Grijalva-Usumacinta |
| C64-383 | Astyanax mexicanus | Astyanax aeneus | Teapa | Grijalva-Usumacinta |
| C64-384 | Astyanax mexicanus | Astyanax aeneus | Teapa | Grijalva-Usumacinta |
| C64-385 | Astyanax mexicanus | Astyanax aeneus | Teapa | Grijalva-Usumacinta |
| C64-386 | Astyanax mexicanus | Astyanax aeneus | Teapa | Grijalva-Usumacinta |
| C64-387 | Astyanax mexicanus | Astyanax aeneus | Teapa | Grijalva-Usumacinta |
| C64-388 | Astyanax mexicanus | Astyanax aeneus | Teapa | Grijalva-Usumacinta |
| C64-389 | Astyanax mexicanus | Astyanax aeneus | Teapa | Grijalva-Usumacinta |
| C64-390 | Astyanax mexicanus | Astyanax aeneus | Teapa | Grijalva-Usumacinta |
| C64-391 | Astyanax mexicanus | Astyanax aeneus | Teapa | Grijalva-Usumacinta |
| C64-392 | Astyanax mexicanus | Astyanax aeneus | Teapa | Grijalva-Usumacinta |
| C64-393 | Astyanax mexicanus | Astyanax aeneus | Teapa | Grijalva-Usumacinta |
| C64-394 | Astyanax mexicanus | Astyanax aeneus | Teapa | Grijalva-Usumacinta |
| C64-395 | Astyanax mexicanus | Astyanax aeneus | Teapa | Grijalva-Usumacinta |
| C64-396 | Astyanax mexicanus | Astyanax aeneus | Teapa | Grijalva-Usumacinta |
| C64-397 | Astyanax mexicanus | Astyanax aeneus | Teapa | Grijalva-Usumacinta |
| C64-398 | Astyanax mexicanus | Astyanax aeneus | Teapa | Grijalva-Usumacinta |
| C64-399 | Astyanax mexicanus | Astyanax aeneus | Teapa | Grijalva-Usumacinta |
| C64-400 | Astyanax mexicanus | Astyanax aeneus | Teapa | Grijalva-Usumacinta |
| C65-401 | Astyanax mexicanus | Astyanax aeneus | Teapa | Grijalva-Usumacinta |
| C65-402 | Astyanax mexicanus | Astyanax aeneus | Tapijulapa | Grijalva-Usumacinta |
| C65-403 | Astyanax mexicanus | Astyanax aeneus | Tapijulapa | Grijalva-Usumacinta |
| C65-404 | Astyanax mexicanus | Astyanax aeneus | Tapijulapa | Grijalva-Usumacinta |
| C65-405 | Astyanax mexicanus | Astyanax aeneus | Tapijulapa | Grijalva-Usumacinta |
| C65-406 | Astyanax mexicanus | Astyanax aeneus | Tapijulapa | Grijalva-Usumacinta |
| C65-407 | Astyanax mexicanus | Astyanax aeneus | Tapijulapa | Grijalva-Usumacinta |
| C65-408 | Astyanax mexicanus | Astyanax aeneus | Tapijulapa | Grijalva-Usumacinta |
| C65-409 | Astyanax mexicanus | Astyanax aeneus | Tapijulapa | Grijalva-Usumacinta |
| C65-410 | Astyanax mexicanus | Astyanax aeneus | Tapijulapa | Grijalva-Usumacinta |
| C65-411 | Astyanax mexicanus | Astyanax aeneus | Tapijulapa | Grijalva-Usumacinta |
| C65-412 | Astyanax mexicanus | Astyanax aeneus | Tapijulapa | Grijalva-Usumacinta |
| C65-413 | Astyanax mexicanus | Astyanax aeneus | Tapijulapa | Grijalva-Usumacinta |
| C65-414 | Astyanax mexicanus | Astyanax aeneus | Tapijulapa | Grijalva-Usumacinta |
| C65-415 | Astyanax mexicanus | Astyanax aeneus | Tapijulapa | Grijalva-Usumacinta |
| C65-416 | Astyanax mexicanus | Astyanax aeneus | Tapijulapa | Grijalva-Usumacinta |
| C65-417 | Astyanax mexicanus | Astyanax aeneus | Tapijulapa | Grijalva-Usumacinta |
| C65-418 | Astyanax mexicanus | Astyanax aeneus | Tapijulapa | Grijalva-Usumacinta |
| C65-419 | Astyanax mexicanus | Astyanax aeneus | Tapijulapa | Grijalva-Usumacinta |
| C65-420 | Astyanax mexicanus | Astyanax aeneus | Tapijulapa | Grijalva-Usumacinta |
| C65-421 | Astyanax mexicanus | Astyanax aeneus | Tapijulapa | Grijalva-Usumacinta |
| C65-422 | Astyanax mexicanus | Astyanax aeneus | Tapijulapa | Grijalva-Usumacinta |
| C65-423 | Astyanax mexicanus | Astyanax aeneus | Tapijulapa | Grijalva-Usumacinta |
| C65-424 | Astyanax mexicanus | Astyanax aeneus | Tapijulapa | Grijalva-Usumacinta |
| C65-425 | Astyanax mexicanus | Astyanax aeneus | Tapijulapa | Grijalva-Usumacinta |
| C65-426 | Astyanax mexicanus | Astyanax aeneus | Tapijulapa | Grijalva-Usumacinta |
| C65-427 | Astyanax mexicanus | Astyanax aeneus | Tapijulapa | Grijalva-Usumacinta |
| C65-428 | Astyanax mexicanus | Astyanax aeneus | Tapijulapa | Grijalva-Usumacinta |
| C65-429 | Astyanax mexicanus | Astyanax aeneus | Río Tzendales | Grijalva-Usumacinta |
| C65-430 | Astyanax mexicanus | Astyanax aeneus | Río Tzendales | Grijalva-Usumacinta |
| C65-431 | Astyanax mexicanus | Astyanax aeneus | Río Tzendales | Grijalva-Usumacinta |
| C65-432 | Astyanax mexicanus | Astyanax aeneus | Río Tzendales | Grijalva-Usumacinta |
| C65-433 | Astyanax mexicanus | Astyanax aeneus | Río Tzendales | Grijalva-Usumacinta |
| C65-434 | Astyanax mexicanus | Astyanax aeneus | Río Tzendales | Grijalva-Usumacinta |
| C65-435 | Astyanax mexicanus | Astyanax aeneus | Río Tzendales | Grijalva-Usumacinta |
| C65-436 | Astyanax mexicanus | Astyanax aeneus | Río Tzendales | Grijalva-Usumacinta |
| C65-437 | Astyanax mexicanus | Astyanax aeneus | Río Tzendales | Grijalva-Usumacinta |
| C65-438 | Astyanax mexicanus | Astyanax aeneus | Río Tzendales | Grijalva-Usumacinta |
| C65-439 | Astyanax mexicanus | Astyanax aeneus | Río Tzendales | Grijalva-Usumacinta |
| C65-440 | Astyanax mexicanus | Astyanax aeneus | Río Tzendales | Grijalva-Usumacinta |
| C65-441 | Astyanax mexicanus | Astyanax aeneus | Río Tzendales | Grijalva-Usumacinta |
| C65-442 | Astyanax mexicanus | Astyanax aeneus | Río Tzendales | Grijalva-Usumacinta |
| C65-443 | Astyanax mexicanus | Astyanax aeneus | Río Tzendales | Grijalva-Usumacinta |
| C65-444 | Astyanax mexicanus | Astyanax aeneus | Río Tzendales | Grijalva-Usumacinta |
| C65-445 | Astyanax mexicanus | Astyanax aeneus | Río Tzendales | Grijalva-Usumacinta |
| C65-446 | Astyanax mexicanus | Astyanax aeneus | Río Tzendales | Grijalva-Usumacinta |
| C65-447 | Astyanax mexicanus | Astyanax aeneus | Río Tzendales | Grijalva-Usumacinta |
| C65-448 | Astyanax mexicanus | Astyanax aeneus | Río Tzendales | Grijalva-Usumacinta |
| C65-449 | Astyanax mexicanus | Astyanax aeneus | Río Tzendales | Grijalva-Usumacinta |
| C65-450 | Astyanax mexicanus | Astyanax aeneus | Río Tzendales | Grijalva-Usumacinta |
| C65-451 | Astyanax mexicanus | Astyanax aeneus | Río Tzendales | Grijalva-Usumacinta |
| C65-452 | Astyanax mexicanus | Astyanax aeneus | Río Tzendales | Grijalva-Usumacinta |
| C65-453 | Astyanax mexicanus | Astyanax aeneus | Río Tzendales | Grijalva-Usumacinta |
| C65-454 | Astyanax mexicanus | Astyanax aeneus | Río Tzendales | Grijalva-Usumacinta |
| C65-455 | Astyanax mexicanus | Astyanax aeneus | Río Tzendales | Grijalva-Usumacinta |
| C65-456 | Astyanax mexicanus | Astyanax aeneus | Río Tzendales | Grijalva-Usumacinta |
| C65-457 | Astyanax mexicanus | Astyanax aeneus | Río Tzendales | Grijalva-Usumacinta |
| C65-458 | Astyanax mexicanus | Astyanax aeneus | Río Tzendales | Grijalva-Usumacinta |
| C65-459 | Astyanax mexicanus | Astyanax aeneus | Río Tzendales | Grijalva-Usumacinta |
| C65-460 | Astyanax mexicanus | Astyanax aeneus | Río Tzendales | Grijalva-Usumacinta |
| C65-461 | Astyanax mexicanus | Astyanax aeneus | Río Tzendales | Grijalva-Usumacinta |
| C65-462 | Astyanax mexicanus | Astyanax aeneus | Río Tzendales | Grijalva-Usumacinta |
| C65-463 | Astyanax mexicanus | Astyanax aeneus | Río Tzendales | Grijalva-Usumacinta |
| C65-464 | Astyanax mexicanus | Astyanax aeneus | Río Tzendales | Grijalva-Usumacinta |
| C65-465 | Astyanax mexicanus | Astyanax aeneus | Río Tzendales | Grijalva-Usumacinta |
| C65-466 | Astyanax mexicanus | Astyanax aeneus | Río Tzendales | Grijalva-Usumacinta |
| C65-467 | Astyanax mexicanus | Astyanax aeneus | Río Tzendales | Grijalva-Usumacinta |
| C65-468 | Astyanax mexicanus | Astyanax aeneus | Río Tzendales | Grijalva-Usumacinta |
| C65-469 | Astyanax mexicanus | Astyanax aeneus | Río Tzendales | Grijalva-Usumacinta |
