# Supplementary material for: Genetic structure and historical and contemporary gene flow of Astyanaxmexicanus in the Gulf of Mexico slope: a microsatellite-based analysis
Source: PeerJ. 2021 Feb 25;9:e10784. doi: 10.7717/peerj.10784 (PMC7916531; doi:10.7717/peerj.10784)
Supplement: Supplemental Information 2 [file peerj-09-10784-s002.docx]

Table S2. Primers and PCR conditions utilized in the amplification of the 10 loci microsatellites.

| **Locus** | **Accession no.** | **Primer sequences (5´-3´)** | **PCR Conditions** | **Amplification Program** |
| --- | --- | --- | --- | --- |
| Ast02^a^ | AY178849 | F-GAGGGTCTGATGTACTGAGA R-CGAACCCGAGAAACTG | Buffer 1.0x MgCl_2_ 2.5.mM dNTPs 0.2mM Primers 0.4μM each Taq Pol 1.0 U | 95°C/5min 95°C/1min 30c 57°C/30s …… .72°C/30s 72°C/5min |
| Ast09^a^ | AY178851 | F-TCCACAATAGACTCGGAAAGAATC R-GACAAAAGAGGCTTCCATGAACT | Buffer 1.0xMgCl_2_ 2.5.mM dNTPs 0.2mM Primers 0.4μM each Taq Pol 1.0 | 95°C/5min95°C/1min 30c 60°C/30s …… .72°C/30s 72°C/5min |
| Ast10^a^ | AY178852 | F-ACAGAAACACGTTGATGTATGGC R-CAAACTGACTGACTGAGTGAGC | Buffer 1.0x MgCl_2_ 2.5.mM dNTPs 0.2mM Primers 0.4μM each Taq Pol 1.0 U | 95°C/5min 95°C/1min 30c 60°C/30s …… .72°C/30s 72°C/5min |
| Am2b^b^ | BV678705 | F-TTTCCAATATTGCCCAGCC R-GACTGACCTCCCACAAGGG | Buffer 1.0x MgCl_2_ 2.0.mM dNTPs 0.2mM Primers 0.4μM each Taq Pol 1.0 U | 95°C/5min 95°C/1min 30c 53°C/30s …… .72°C/30s 72°C/5min |
| Am214d^b^ | BV678864 | F-CTGCAAATGAACACTCAGCG R-TCCTCCTCGTCACATATAGGC | Buffer 1.0x MgCl_2_ 2.5.mM dNTPs 0.2mM Primers 0.4μM each Taq Pol 1.0 U | 95°C/5min 95°C/1min 30c 56°C/30s …… .72°C/30s 72°C/5min |
| Am241b^b^ | BV678952 | F-CATAATGTAAAGTGCGGGGG R-TCACCTCCTATTAGCTCACGC | Buffer 1.0xMgCl_2_ 2.5.mM dNTPs 0.2mM Primers 0.4μM each Taq Pol 1.0 U | 95°C/5min95°C/1min 30c 52°C/30s …… .72°C/30s 72°C/5min |
| Am145a^b^ | BV678816 | F-TTTTCAGGGTCCAGTAACGC R-CCAGACCATCAAGAAGACGG | Buffer 1.0xMgCl_2_ 2.5.mM dNTPs 0.2mM Primers 0.4μM each Taq Pol 1.0 U | 95°C/5min95°C/1min 30c 52°C/30s …… .72°C/30s 72°C/5min |
| Am26c^b^ | BV678725 | F-ATGTGGTGAAACTGATTGGC R-ACAGCACCTCCCTCGACCC | Buffer 1.0xMgCl_2_ 2.5.mM dNTPs 0.2mM Primers 0.4μM each Taq Pol 1.0 U | 95°C/5min95°C/1min 30c 52°C/30s …… .72°C/30s 72°C/5min |
| Am122b^b^ | BV678784 | F-ACAACATCAGCAAGTTCCCC R-ATGGCACTCGATTACAGCG | Buffer 1.0xMgCl_2_ 2.5.mM dNTPs 0.2mM Primers 0.4μM each Taq Pol 1.0 U | 95°C/5min95°C/1min 30c 55°C/30s …… .72°C/30s 72°C/5min |
| Am106b^b^ | BV678756 | F-CTCACGAGTCAGCATTTCCC R-TCAACTTGCATAGACAGGGC | Buffer 1.0xMgCl_2_ 2.5.mM dNTPs 0.2mM Primers 0.4μM each Taq Pol 1.0 U | 95°C/5min95°C/1min 30c 55°C/30s …… .72°C/30s 72°C/5min |

Primers from:

a: Strecker, U. 2003. Polymorphic microsatellites isolated from the cave fish *Astyanax fasciatus*. Molecular Ecology Notes 3:150-151.

b: Protas, E. M., C. Hersey, D. Kochanek, Y. Zhou, H. Wilkens, W.R. Jeffery, L.I. Zon, R. Borowsky y C. Tabin. 2006. Genetic analysis of cavefish reveals molecular convergence in the evolution of albinism. Nature Genetics, 38(1): 107-111.
