# Supplementary material for: Genetic structure and historical and contemporary gene flow of Astyanaxmexicanus in the Gulf of Mexico slope: a microsatellite-based analysis
Source: PeerJ. 2021 Feb 25;9:e10784. doi: 10.7717/peerj.10784 (PMC7916531; doi:10.7717/peerj.10784)
Supplement: Supplemental Information 3 [file peerj-09-10784-s003.docx]

Table S3. Genetic variability estimated by 10 microsatellite loci of *Astyanax* spp. Sample size (N), number of alleles (Na), effective alleles (Nae), allelic richness (A_R_) with rarefaction approach, observed heterozygosity (Ho), unbiased expected heterozygosity (uHe) and fixation index (F).

| **Population** | **Estimator** | **Ast09** | **Ast10** | **Ast02** | **Am2b** | **Am214d** | **Am241b** | **Am145a** | **Am26c** | **Am122b** | **Am106b** | **Average** |
| --- | --- | --- | --- | --- | --- | --- | --- | --- | --- | --- | --- | --- |
|  | N | 26 | 26 | 26 | 25 | 25 | 26 | 26 | 26 | 26 | 26 | 25.8 |
| **CC** | Na | 6 | 4 | 4 | 9 | 8 | 3 | 5 | 7 | 3 | 7 | 5.6 |
|  | Nae | 2.042 | 1.219 | 2.508 | 3.521 | 3.125 | 1.985 | 2.537 | 4.433 | 1.431 | 3.159 | 2.596 |
|  | A_R_ | 5.1587 | 3.2285 | 3.8254 | 7.1865 | 6.3417 | 2.5769 | 4.7291 | 6.4361 | 2.9628 | 5.5553 | 4.8 |
|  | H*o* | 0.462 | 0.192 | 0.5 | 0.68 | 0.52 | 0.462 | 0.5 | 0.692 | 0.346 | 0.654 | 0.5 |
|  | uH*e* | 0.520 | 0.183 | 0.613 | 0.731 | 0.694 | 0.506 | 0.618 | 0.790 | 0.307 | 0.697 | 0.566 |
|  | F | 0.096 | -0.07 | 0.169 | 0.05 | 0.235 | 0.07 | 0.175 | 0.106 | -0.15 | 0.043 | 0.072 |
|  | N | 30 | 30 | 30 | 30 | 30 | 29 | 30 | 30 | 30 | 30 | 29.9 |
| **SF** | Na | 4 | 6 | 7 | 7 | 13 | 11 | 5 | 7 | 6 | 7 | 7.3 |
|  | Nae | 1.619 | 1.579 | 1.278 | 2.791 | 6.569 | 4.485 | 2.12 | 3.333 | 3.136 | 4.444 | 3.135 |
|  | A_R_ | 3.5081 | 4.2519 | 4.2542 | 5.2281 | 10.567 | 9.1047 | 4.4823 | 5.7617 | 4.5 | 6.4767 | 5.81 |
|  | H*o* | 0.4 | 0.267 | 0.2 | 0.767 | 0.833 | 0.586 | 0.4 | 0.633 | 0.7 | 0.8 | 0.558 |
|  | uH*e* | 0.389 | 0.373 | 0.221 | 0.653 | 0.862 | 0.791 | 0.537 | 0.712 | 0.693 | 0.788 | 0.602 |
|  | F | -0.047 | 0.273 | 0.079 | -0.195 | 0.017 | 0.246 | 0.243 | 0.095 | -0.028 | -0.032 | 0.065 |
|  | N | 64 | 64 | 64 | 64 | 64 | 64 | 64 | 64 | 64 | 64 | 64 |
| **GV-TR** | Na | 9 | 10 | 23 | 13 | 11 | 16 | 10 | 9 | 6 | 19 | 12.6 |
|  | Nae | 2.256 | 3.664 | 11.237 | 4.171 | 5.007 | 8.623 | 5.282 | 3. 114 | 1.571 | 6.059 | 5.098 |
|  | A_R_ | 4.5806 | 6.9047 | 13.9231 | 8.262 | 7.5642 | 10.9655 | 7.7336 | 5.9669 | 2.937 | 11.0915 | 7.99 |
|  | H*o* | 0.469 | 0.75 | 0.813 | 0.672 | 0.672 | 0.891 | 0.594 | 0.547 | 0.359 | 0.75 | 0.652 |
|  | uH*e* | 0.561 | 0.733 | 0.918 | 0.766 | 0.807 | 0.891 | 0.817 | 0.684 | 0.366 | 0.842 | 0.738 |
|  | F | 0.158 | -0.032 | 0.108 | 0.116 | 0.16 | -0.007 | 0.268 | 0.194 | 0.011 | 0.102 | 0.108 |
|  | N | 81 | 81 | 81 | 81 | 81 | 81 | 81 | 81 | 81 | 79 | 80.6 |
| **AL-** | Na | 10 | 12 | 19 | 18 | 20 | 16 | 12 | 11 | 13 | 15 | 14.6 |
| **LCA-SLP** | Nae | 2.319 | 3.775 | 11.8 | 8.673 | 7.051 | 7.72 | 7.155 | 8.148 | 4.42 | 6.722 | 6.778 |
|  | A_R_ | 5.7123 | 8.5572 | 12.9566 | 11.2184 | 11.8553 | 9.8179 | 8.9552 | 9.1371 | 8.6275 | 9.2089 | 9.6 |
|  | H*o* | 0.481 | 0.617 | 0.605 | 0.741 | 0.788 | 0.825 | 0.889 | 0.8 | 0.679 | 0.671 | 0.709 |
|  | uH*e* | 0.572 | 0.740 | **0.921*** | 0.890 | 0.864 | 0.876 | 0.866 | 0.883 | 0.779 | 0.857 | 0.825 |
|  | F | 0.154 | 0.16 | 0.339 | 0.163 | 0.094 | 0.052 | -0.033 | 0.088 | 0.122 | 0.212 | 0.135 |
|  | N | 15 | 15 | 15 | 15 | 15 | 15 | 15 | 15 | 15 | 15 | 15 |
| **ML** | Na | 1 | 1 | 2 | 1 | 1 | 1 | 1 | 3 | 1 | 1 | 1.3 |
|  | Nae | 1 | 1 | 1.069 | 1 | 1 | 1 | 1 | 1.744 | 1 | 1 | 1.081 |
|  | A_R_ | 1 | 1 | 2 | 1 | 1 | 1 | 1 | 3 | 1 | 1 | 1.3 |
|  | H*o* | 0 | 0 | 0.067 | 0 | 0 | 0 | 0 | 0.267 | 0 | 0 | 0.033 |
|  | uH*e* | 0.000 | 0.000 | 0.067 | 0.000 | 0.000 | 0.000 | 0.000 | 0.441 | 0.000 | 0.000 | 0.051 |
|  | F | ---- | ---- | -0.03 | ---- | ---- | ---- | ---- | 0.375 | ---- | ---- | 0.172 |
|  | N | 44 | 44 | 44 | 44 | 44 | 44 | 44 | 44 | 44 | 44 | 44 |
| **PCH** | Na | 1 | 2 | 2 | 4 | 2 | 1 | 3 | 4 | 2 | 1 | 2.2 |
|  | Nae | 1 | 1.095 | 1.172 | 1.234 | 1.071 | 1 | 1.606 | 1.506 | 1.252 | 1 | 1.081 |
|  | A_R_ | 1 | 1.8181 | 1.9526 | 2.9428 | 1.7188 | 1 | 2.7186 | 3.696 | 1.9884 | 1 | 1.98 |
|  | H*o* | 0 | 0.091 | 0.159 | 0.182 | 0.023 | 0 | 0.341 | 0.227 | 0.227 | 0 | 0.033 |
|  | uH*e* | 0.000 | 0.088 | 0.148 | 0.192 | 0.067 | 0.000 | 0.382 | 0.340 | 0.204 | 0.000 | 0.142 |
|  | F | ---- | -0.048 | -0.086 | 0.041 | 0.665 | ---- | 0.097 | 0.324 | -0.128 | ---- | 0.172 |
|  | N | 35 | 34 | 35 | 34 | 34 | 35 | 35 | 35 | 35 | 35 | 34.7 |
| **SAB-TIN** | Na | 1 | 5 | 5 | 1 | 3 | 1 | 6 | 3 | 3 | 2 | 3 |
|  | Nae | 1 | 1.592 | 3.059 | 1 | 1.883 | 1 | 1.698 | 2.125 | 2.731 | 1.059 | 1.715 |
|  | A_R_ | 1 | 3.9609 | 4.1056 | 1 | 2.9094 | 1 | 4.694 | 2.9958 | 2.9997 | 1.677 | 2.63 |
|  | H*o* | 0 | 0.147 | 0.371 | 0 | 0.235 | 0 | 0.229 | 0.257 | 0.686 | 0 | 0.193 |
|  | uH*e* | 0.000 | 0.378 | **0.682*** | 0.000 | 0.476 | 0.000 | 0.417 | 0.537 | 0.643 | 0.056 | 0.319 |
|  | F | ---- | 0.605 | 0.448 | ---- | 0.498 | ---- | 0.444 | 0.514 | -0.082 | 1 | 0.49 |
|  | N | 30 | 30 | 30 | 29 | 30 | 30 | 30 | 30 | 30 | 30 | 29.9 |
| **V** | Na | 3 | 5 | 10 | 13 | 9 | 13 | 12 | 8 | 9 | 13 | 9.5 |
|  | Nae | 1.867 | 1.37 | 4.749 | 6.95 | 4.639 | 7.229 | 7.627 | 6.122 | 4.639 | 6.569 | 5.176 |
|  | A_R_ | 2.9973 | 3.8252 | 8.5799 | 10.1705 | 8.0574 | 10.2949 | 9.9729 | 7.4937 | 8.0593 | 10.5558 | 8 |
|  | H*o* | 0.6 | 0.233 | 0.567 | 0.759 | 0.633 | 0.833 | 0.8 | 0.833 | 0.667 | 0.867 | 0.679 |
|  | uH*e* | 0.472 | 0.275 | 0.803 | 0.871 | 0.798 | 0.876 | 0.884 | 0.851 | 0.798 | 0.862 | 0.749 |
|  | F | -0.292 | 0.136 | 0.282 | 0.114 | 0.193 | 0.033 | 0.079 | 0.004 | 0.15 | -0.022 | 0.067 |
|  | N | 26 | 26 | 26 | 26 | 26 | 26 | 26 | 26 | 26 | 26 | 26 |
| **CAT** | Na | 4 | 11 | 18 | 13 | 12 | 14 | 6 | 8 | 10 | 14 | 11 |
|  | Nae | 1.865 | 7.116 | 7.47 | 6.66 | 7.86 | 8.837 | 4.39 | 5.408 | 4.404 | 9.197 | 6.32 |
|  | A_R_ | 3.9447 | 9.527 | 13.9277 | 10.9367 | 10.6455 | 11.2345 | 5.8931 | 7.3023 | 8.2425 | 11.6743 | 9.33 |
|  | H*o* | 0.423 | 0.577 | 0.885 | 0.923 | 0.846 | 0.846 | 0.692 | 0.654 | 0.769 | 0.577 | 0.719 |
|  | uH*e* | 0.473 | **0.876*** | 0.883 | 0.867 | 0.890 | 0.904 | 0.787 | 0.831 | 0.788 | **0.909*** | 0.821 |
|  | F | 0.088 | 0.329 | -0.021 | -0.086 | 0.031 | 0.046 | 0.103 | 0.198 | 0.005 | 0.353 | 0.104 |
|  | N | 118 | 118 | 118 | 118 | 118 | 118 | 118 | 118 | 118 | 118 | 118 |
| **TE-TA** | Na | 14 | 11 | 23 | 20 | 17 | 18 | 13 | 15 | 20 | 15 | 16.6 |
| **RTZ** | Nae | 6.545 | 1.965 | 10.505 | 9.173 | 8.126 | 9.669 | 5.871 | 10.246 | 9.785 | 7.689 | 7.957 |
|  | A_R_ | 9.0652 | 6.2625 | 12.474 | 11.1837 | 11.09 | 12.1997 | 8.1667 | 10.7935 | 10.9364 | 10.1825 | 10.24 |
|  | H*o* | 0.805 | 0.483 | 0.856 | 0.814 | 0.822 | 0.89 | 0.669 | 0.822 | 0.763 | 0.864 | 0.779 |
|  | H*e* | 0.851 | 0.493 | 0.909 | 0.895 | 0.881 | 0.900 | 0.833 | 0.906 | 0.902 | 0.874 | 0.844 |
|  | F | 0.05 | 0.016 | 0.054 | 0.087 | 0.063 | 0.008 | 0.193 | 0.089 | 0.15 | 0.006 | 0.072 |

* Hardy-Weinberg disequilibrium after the Bonferroni adjustment with P <0.001. For the meaning of the acronyms, see Table 1.
