# Supplementary material for: Genetic structure and historical and contemporary gene flow of Astyanaxmexicanus in the Gulf of Mexico slope: a microsatellite-based analysis
Source: PeerJ. 2021 Feb 25;9:e10784. doi: 10.7717/peerj.10784 (PMC7916531; doi:10.7717/peerj.10784)
Supplement: Supplemental Information 4 — Sample size (N), number of alleles (Na), effective alleles (Nae), allelic richness (R), observed heterozygosity (Ho), expected heterozygosity (He) and fixation index (F). (*) Hardy-Weinberg disequilibrium after the Bonferroni adjustment with P < 0.001. For the meaning of the acronyms, see Table 1. [file peerj-09-10784-s004.docx]

Table S4. Frequency of null alleles per locus and population. Estimation conducted according to the Brookfield 1 estimator (B) in the MICRO-CHECKER (Van Oosterhout et al., 2004) and the EM algorithm in FreeNA (Chapuis and Estoup, 2007).

| **Population** | **Estimator** | **Ast09** | **Ast10** | **Ast02** | **Am2b** | **Am214d** | **Am241b** | **Am145a** | **Am26c** | **Am122b** | **Am106b** |
| --- | --- | --- | --- | --- | --- | --- | --- | --- | --- | --- | --- |
| CC | B  EM | ------- 0.0393* | -------  0.0000 | -------  0.0842 | -------  0.0415 | 0.1131  0.0841 | -------  0.0149 | -------  0.0678 | -------  0.0390 | -------  0.0000 | -------  0.0000 |
| SF | B  EM | -------  0.000 | -------  0.0418 | -------  0.0000 | ------- 0.0000 | -------  0.0117 | 0.0811*  0.1096 | -------  0.0793 | -------  0.0334 | -------  0.0000 | ------- 0.0000 |
| GV-TR | B  EM | 0.1048  0.0747 | -------  0.0068 | 0.0412  0.0387 | ------- 0.0177 | 0.0760  0.0727 | ------- 0.0152 | 0.0880*  0.1178 | 0.0823  0.0524 | ------- 0.0000 | ------- 0.0167 |
| AL-LCA-SLP | B  EM | -------- 0.0278 | 0.0776 0.0742 | 0.0441  0.1602 | 0.0513  0.0731 | ------- 0.0489 | ------- 0.0164 | ------- 0.0040 | ------- 0.0341 | 0.0728*  0.0726 | 0.0601*  0.0925 |
| ML | B  EM | -------0.0010 | --------  0.0010 | --------  0.0000 | -------  0.0010 | -------  0.0010 | -------  0.0010 | -------  0.0010 | ------- 0.0008 | ------- 0.0010 | ------- 0.0010 |
| PCH | B  EM | ------- 0.0010 | -------- 0.0000 | -------- 0.0000 | ------- 0.0000 | ------- 0.0936 | ------- 0.0010 | ------- 0.0317 | ------- 0.0000 | ------- 0.0000 | ------- 0.0010 |
| SAB-TIN | B  EM | -------  0.0010 | ------- 0.1772 | 0.1366* 0.1780 | ------- 0.0010 | ------- 0.1674 | ------- 0.0010 | ------- 0.1347 | ------- 0.1491 | ------- 0.0000 | ------- 0.1148 |
| V | B  EM | ------- 0.0000 | ------- 0.0555 | 0.0875 0.1195 | ------- 0.0673 | 0.1017 0.0803 | ------- 0.0032 | ------- 0.0353 | ------- 0.0000 | ------- 0.0528 | ------- 0.0000 |
| CAT | B  EM | ------- 0.0000 | 0.1138*  0.1584 | ------- 0.0000 | ------- 0.0000 | ------- 0.0264 | ------- 0.0008 | ------- 0.0521 | 0.0696  0.0607 | ------- 0.0000 | 0.1443*  0.1651 |
| TE-TA-RTZ | B  EM | ------- 0.0020 | ------- 0.0000 | ------- 0.0196 | -------- 0.0295 | ------- 0.0202 | ------- 0.0185 | 0.0702 0.0853 | ------- 0.0394 | 0.0464 0.0676 | ------- 0.0130 |

* denotes significant result (*P* ≤ 0.05). For the meaning of acronyms, see Table 1. -----Null alleles not detected.
