# Supplementary material for: Genetic structure and historical and contemporary gene flow of Astyanaxmexicanus in the Gulf of Mexico slope: a microsatellite-based analysis
Source: PeerJ. 2021 Feb 25;9:e10784. doi: 10.7717/peerj.10784 (PMC7916531; doi:10.7717/peerj.10784)
Supplement: Supplemental Information 5 — Estimation conducted according to the Brookfield 1 estimator (B) in the MICRO-CHECKER (Van (Oosterhout, Wills & Hutchinson, 2004) and the EM algorithm in FreeNA (Chapuis & Estoup, 2007). * denotes significant result (P ≤ 0.05). For the meaning of acronyms, see Table 1. —–Null alleles not detected. [file peerj-09-10784-s005.docx]

Table S5. Comparison of linkage disequilibrium among pairs of loci in *A. mexicanus*

|  |  |  |  |
| --- | --- | --- | --- |

| loci pair | p-value | loci pair | p-value |
| --- | --- | --- | --- |
| Ast09 & Ast10 | 0.3817 | Ast02 & Am26c | 0.3035 |
| Ast09 & Ast02 | 0.8876 | Am2b & Am26c | 0.5743 |
| Ast10 & Ast02 | 0.2831 | Am214d & Am26c | 0.2513 |
| Ast09 & Am2b | 0.3245 | Am241b & Am26c | 0.0931 |
| Ast10 & Am2b | 0.3348 | Am145a & Am26c | 0.1174 |
| Ast02 & Am2b | 0.9100 | Ast09 & Am122b | 0.4740 |
| Ast09 & Am214d | 0.9855 | Ast10 & Am122b | 0.0522 |
| Ast10 & Am214d | 0.5526 | Ast02 & Am122b | 0.2603 |
| Ast02 & Am214d | 0.9810 | Am2b & Am122b | 0.0199 |
| Am2b & Am214d | 0.4076 | Am214d & Am122b | 0.6938 |
| Ast09 & Am241b | 0.4233 | Am241b & Am122b | 0.1448 |
| Ast10 & Am241b | 0.1424 | Am145a & Am122b | 0.4369 |
| Ast02 & Am241b | 0.9996 | Am26c & Am122b | 0.0650 |
| Am2b & Am241b | 0.5551 | Ast09 & Am106B | 0.0878 |
| Am214d & Am241b | 0.3098 | Ast10 & Am106B | 0.9296 |
| Ast09 & Am145a | 0.1039 | Ast02 & Am106B | 0.9579 |
| Ast10 & Am145a | 0.0639 | Am2b & Am106B | 0.6451 |
| Ast02 & Am145a | 0.9994 | Am214d & Am106B | 0.4691 |
| Am2b & Am145a | 0.2417 | Am241b & Am106B | 0.9490 |
| Am214d & Am145a | 0.8618 | Am145a & Am106B | 0.3298 |
| Am241b & Am145a | 0.6615 | Am26c & Am106B | 0.7076 |
| Ast09 & Am26c | 0.9477 | Am122b & Am106B | 0.5528 |
| Ast10 & Am26c | 0.0256 |  |  |

|  |  |  |  |
| --- | --- | --- | --- |
|  |  |  |  |
|  |  |  |  |
|  |  |  |  |
|  |  |  |  |
|  |  |  |  |
|  |  |  |  |
|  |  |  |  |
|  |  |  |  |
|  |  |  |  |
|  |  |  |  |
|  |  |  |  |
|  |  |  |  |
|  |  |  |  |
|  |  |  |  |
|  |  |  |  |
|  |  |  |  |
|  |  |  |  |
|  |  |  |  |
|  |  |  |  |
|  |  |  |  |
|  |  |  |  |
|  |  |  |  |
