# Supplementary material for: Genetic structure and historical and contemporary gene flow of Astyanaxmexicanus in the Gulf of Mexico slope: a microsatellite-based analysis
Source: PeerJ. 2021 Feb 25;9:e10784. doi: 10.7717/peerj.10784 (PMC7916531; doi:10.7717/peerj.10784)
Supplement: Supplemental Information 6 [file peerj-09-10784-s006.docx]

Table S6. Pairwise F_ST_ between *A. mexicanus* locations using and not using ENA algorithm with FreeNA.

| **All loci** | |
| --- | --- |
| **Fst not using ENA** | **Fst not using ENA** |
| 0.264312 | 0.26159 |
| **Bootstrap resampling over loci 95% confidence interval** | |
| 0.228405 | 0.224525 |
| 0.309007 | 0.305796 |

|  | **Per locus** | |
| --- | --- | --- |
| **Locus** | **Fst not using ENA** | **Fst not using ENA** |
| 1 | 0.352492 | 0.353243* |
| 2 | 0.427306 | 0.419333 |
| 3 | 0.220837 | 0.221221 |
| 4 | 0.240031 | 0.243327 |
| 5 | 0.225407 | 0.212534 |
| 6 | 0.273083 | 0.273921* |
| 7 | 0.234902 | 0.230129* |
| 8 | 0.174466 | 0.165282 |
| 9 | 0.256044 | 0.261662* |
| 10 | 0.263187 | 0.256983* |

^*Null alleles detected^
