# Supplementary figures and images for: Genetic structure and historical and contemporary gene flow of Astyanaxmexicanus in the Gulf of Mexico slope: a microsatellite-based analysis
Source: PeerJ. 2021 Feb 25;9:e10784. doi: 10.7717/peerj.10784 (PMC7916531; doi:10.7717/peerj.10784)

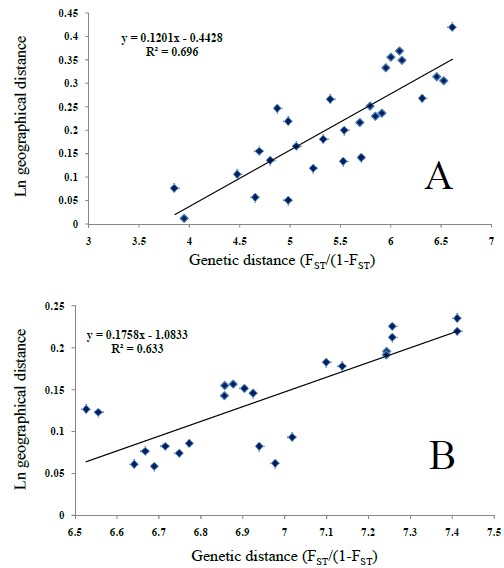

Supplement: Supplemental Information 7 — (A) only locations to the north of the Trans- Mexican Volcanic Belt (Cuatro Ciénegas, San Fernando, Garza Valdez, Troncones, Arroyo Lagartos, La Cañada, Huichihuayan y el Zapotal). (B) all surface locations, except for Catemaco. y = linear regression function and R2= determination coefficient. [file peerj-09-10784-s007.jpg]

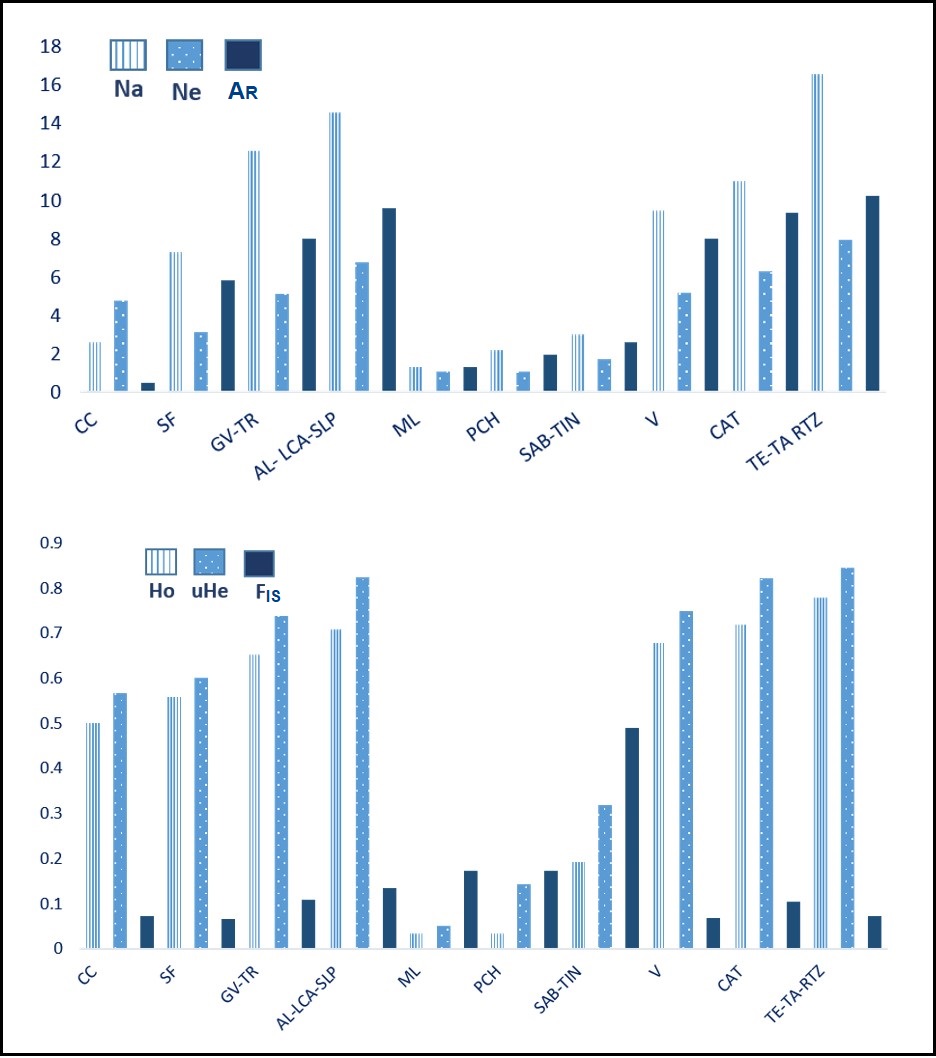

Supplement: Supplemental Information 8 [file peerj-09-10784-s008.jpg]
